# Evaluating the anticancer properties of VAF: a novel folate-α-tocopherol conjugate against lung cancer cells

**DOI:** 10.1186/s12885-025-14954-8

**Published:** 2025-09-16

**Authors:** Shams H. Abdel-Hafez, Shaimaa M. I. Alexeree

**Affiliations:** 1 Department of Chemistry, Faculty of Science, Assiut University, Assiut, 71516 Egypt; 2 Department of Laser application in Metrology, Photochemistry and Photobiology, Agriculture National Institute of Laser Enhanced Science, Cairo University, Giza, 12613 Egypt

**Keywords:** α-tocopherol, Folic acid, Folate conjugated α-tocopherol, Wi-38, A549, lung cancer cells

## Abstract

**Purpose:**

In this study, a new chemical folate conjugated to α-tocopherol (VAF) is introduced. The main goal is to investigate the potential of VAF as a strong anticancer agent. This improves the drug-targeting delivery system and lessens the negative effects associated with traditional chemotherapy.

**Procedure:**

To synthesize VAF, α-tocopherol, and folic acid were linked by a peptide bond, creating an amphiphilic structure that allowed for self-assembly in aqueous conditions. With an emphasis on evaluating VAF’s capacity to target folate receptors, In vitro experiments were carried out utilizing A549 lung cancer cells to assess the anticancer effects of VAF.

**Results:**

VAF was synthesized under mild circumstances, and its characterization was carried out using many analytical techniques, such as FTIR spectroscopy and NMR. In vitro investigations showed that VAF’s improved active targeting via folate receptors results in excellent anticancer effects. Additionally, the cell viability of Wi-38 and A549 was assessed by using confocal laser scanning microscopy (CLSM).

**Conclusion:**

According to the research, VAF reduces the possibility of adverse effects while increasing the efficacy of anticancer treatments. The compound exhibits promising properties that make it a great option for additional research and development in cancer treatments. Research provides important new information about drug-targeting delivery systems and improves a potentially useful method for anticancer drug therapeutic indices.

**Supplementary Information:**

The online version contains supplementary material available at 10.1186/s12885-025-14954-8.

## Introduction

Cancer is a multidimensional and intricate disease that is typified by the uncontrolled growth of aberrant cells, which can arise in nearly any tissue or organ in the body. Unchecked growth can result in the production of tumors that are categorized as benign (non-cancerous) or malignant (cancerous). Cancerous growths can spread to distant locations and infiltrate surrounding tissues. Cancer continues to be a leading cause of morbidity and mortality worldwide. Globally, driving the search for more effective and selective cancer treatment is a challenging undertaking. Moreover, the use of traditional drug delivery systems leads to serious toxic side effects due to these drugs being incapable of identifying tumor cells from healthy cells. The application of targeted treatments, which work only on target cells, has grown significantly in the last few decades [1, 2].

Folic acid and Vitamin E have a significant role in the context of cancer biology, especially due to their involvement in cellular processes that may influence cancer development and progression. Folic acid (Folate) is a water-soluble Vitamin B9. The body uses folate to make DNA repair, and methylation processes that are vital for maintaining genomic stability [3]. Furthermore, Folic Acid (FA) is a targeting agent whose potent binding to folate receptors has made it a promising candidate for targeted medication delivery systems. Curiously, while the majority of normal cells lack folate receptors or have very low quantities of them on their surface, many cancer cells have highly expressed folate receptors. FA could be introduced to increase treatment efficacy and prevent damage to normal tissues [4, 5]. A review of the literature found that folic acid may be incorporated as a ligand in liposome systems through conjugated folic acid/cholesterol, which resulted in highly selective liposome-encapsulated doxorubicin that was more cytotoxic inside tumors [6]. Historically, folic acid is linked to lipids or polymers with polyethylene glycol serving as the linker, which is used as a targeted ligand on several occasions (Folate-PEG) [7–9].

Vitamin E, a fat-soluble antioxidant, is composed of eight natural lipophilic molecules that have a similar molecular structure. Two subgroups comprise them: Tocopherols and Tocotrienols. There are four isoforms in each group (α, γ, β, δ). Nuts and vegetable-based oils provide adequate amounts of Vitamin E, which is a significant food’s antioxidant content [10]. Vitamin E antioxidant is known for its ability to minimize free radical damage and shield cells from oxidative damage, which is a major factor in the development of cancer. Research has shown that vitamin E may inhibit the proliferation of cancer cells and induce apoptosis, although the results are not universally consistent across all cancer types [11, 12]. Historically, It is generally accepted that the primary type of Vitamin E found in dietary supplements is α-Tocopherol. This is responsible for regulating Vitamin A processes within the body. Its anti-inflammatory qualities also shield the skin from detrimental impacts. Moreover, α-Tocopherol creates an intermediate tocopheroxide analog when it interacts with nitrogen dioxide (NO_2_) [13]. Due to α-tocopherol’s trimethylation, the nitrosating agent can only add to the para-position on its chromanol ring. This results in the formation of a highly unstable molecule, which can lead to the formation of hazardous N-nitroso derivatives from amines [14]. Furthermore, the reaction between α-tocopherol and nitrous acid can produce α-tocopherol quinone and nitrogen monoxide gas [15]. This may result in extremely unstable derivatives that can catalyze secondary amine nitrosation. Owing to its great capacity for hydrogen donation, Prooxidant, and dangerous nitro derivatives are among the undesirable side effects of α-tocopherol that can occur [16]. The type of Vitamin E that has been investigated the most for cancer prevention and treatment is α-tocopherol [17].

Lung cancer is the most frequent cancer worldwide. Non-small-cell lung cancer (NSCLC), which includes subtypes like adenocarcinoma, squamous cell carcinoma, and large cell carcinoma, accounts for around 85% of all cases of lung cancer remains one of the leading causes of cancer-related mortality all over the world [2]. So, it is underscoring the urgent need for more effective targeted therapies. A promising molecular target in NSCLC is the folate receptor alpha (FRα), which is highly overexpressed in approximately 74% of lung adenocarcinomas, while being largely absent or minimally expressed in normal tissues [18]. This selective overexpression facilitates the development of folate-conjugated therapeutic agents that can preferentially bind and enter cancer cells via receptor-mediated endocytosis, thereby enhancing drug delivery specificity and reducing systemic toxicity. Moreover, high FRα expression in lung tumors has been correlated with improved prognosis and increased sensitivity to antifolate chemotherapy, further validating its clinical relevance. The α-tocopherol component offers additional therapeutic benefits due to its antioxidant and anticancer properties, which may synergize with folate-mediated targeting to improve efficacy. Taken together, the conjugation of folic acid with α-tocopherol leverages the overexpression of FRα in lung cancer cells to achieve targeted delivery, representing a strategic approach to enhance treatment outcomes in NSCLC patients. Future investigations will explore the efficacy of this conjugate across various FRα-expressing cancer types to expand its therapeutic potential [19, 20].

Conventional drug delivery systems often face significant challenges, including poor selectivity, rapid clearance, systemic toxicity, and limited accumulation at the tumor site, all of which can reduce therapeutic efficacy and increase adverse effects [21, 22]. These limitations have prompted the development of targeted drug delivery strategies aimed at improving specificity and minimizing off-target toxicity. Among these, folate receptor-mediated targeting has gained considerable attention due to the overexpression of folate receptors in various malignancies, including lung cancer [23]. In this context, the design of the folate–α-tocopherol conjugate (VAF) provides a dual advantage: folate enables selective targeting of cancer cells, while α-tocopherol contributes antioxidant and cytotoxic properties. This conjugation strategy aims to enhance intracellular uptake through receptor-mediated endocytosis, increase the local drug concentration within tumor tissues, and thereby improve therapeutic outcomes while minimizing systemic side effects [24]. The integration of targeting ligands with bioactive molecules such as VAF thus represents a promising approach in the development of more efficient and safer cancer therapies.

This work introduces innovative insights into the fabrication and physicochemical characterization of a novel folate-conjugated α-tocopherol (VAF), specifically engineered for targeted cancer therapy. The main purpose is to evaluate the selective cytotoxicity of VAF against A549 lung cancer cells while preserving the viability of healthy lung cells (Wi-38). This work investigates VAF’s potential as a dual-function therapeutic agent, providing accuracy in cancer targeting and reducing off target toxicity, by utilizing the inherent biocompatibility and targeting characteristics of its constituents.

## Materials and methods

### Materials

α-Tocopherol, dicyclohexyl carbodiimide (DCC), n-hydroxy succinamide (NHS), chloroacetamide, and folic acid acquired by the Norwegian University of Science and Technology (NTNU). All materials were purchased from Sigma-Aldrich. Melting points were calculated using a Kofler melting point device. At Taif University, Pye-Unicam SP3-100 equipment was used to measure Fourier transform infrared (FT-IR) analysis (KBr, cm − 1). King Abdul-Aziz University used a Varian (400 MHz) EM 390 USA instrument to record proton NMR spectra in ppm with TMS as a reference.t. A JNM-LA spectrometer operating at 100 MHz was used to record 13 C NMR spectra. A JEOL-JMS-AX 500 was used to measure mass spectra at the National Research Centre in Cairo, Egypt.

### Synthesis and purification of α-tocopherol-folic acid conjugate

The conjugate was synthesized via a two-step procedure: (1) preparation of the amino derivative of vitamin E, followed by (2) carbodiimide-mediated coupling to activated folic acid. Purification was achieved through liquid-liquid extraction and recrystallization.

#### Step 1: synthesis of amino-derivatized vitamin E

**Fig. 1:**
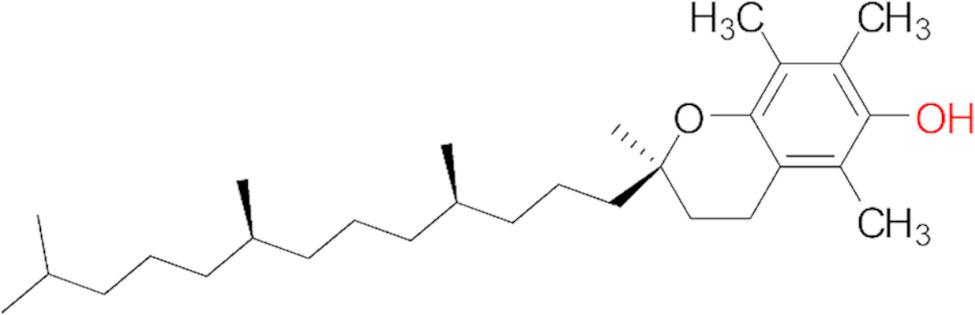
Shows the structure of Vitamin E derivatives (α-Tocopherol)

α-Tocopherol (0.43 g, 1.0 mmol) was dissolved in anhydrous ethanol (20 mL). Sodium hydroxide (0.04 g, 1.0 mmol) and chloroacetamide (0.093 g, 1.0 mmol) were added sequentially. The mixture was stirred at 25 °C for 48 h under inert atmosphere. Workup and Purification: Ethyl acetate (30 mL) and brine (10 mL) were added to the reaction mixture. The aqueous layer was extracted with ethyl acetate (2 × 15 mL). The combined organic extracts were washed with brine (2 × 10 mL) and water (1 × 10 mL). The organic phase was dried over anhydrous Na₂SO₄ and concentrated *in vacuo* to yield the amino derivative as a viscous oil see Fig. 1.

#### Step 2: conjugation with folic acid

**Fig. 2:**
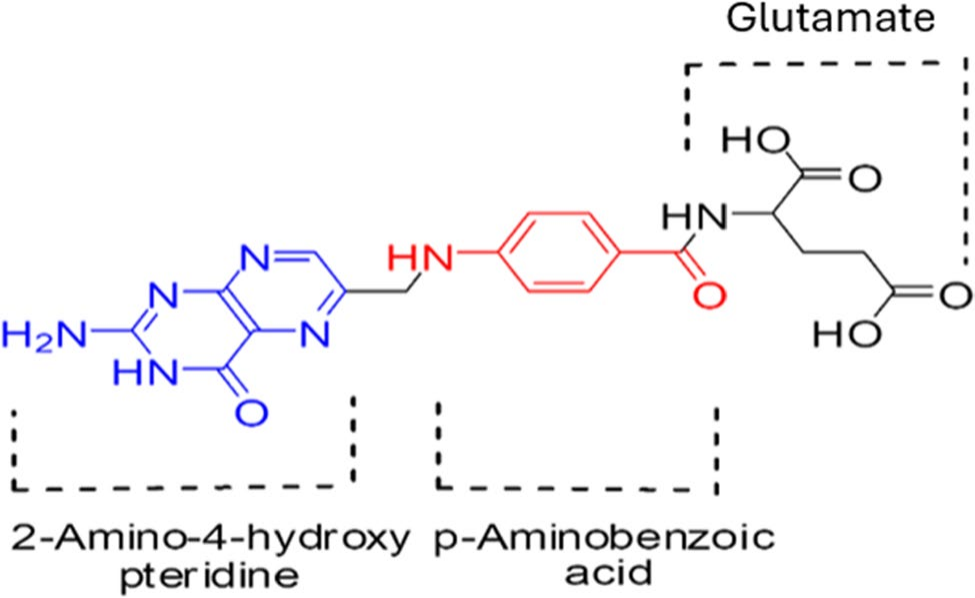
Shows the folic acid structure

Folic acid (0.44 g, 1.0 mmol) was dissolved in dry DMSO (10 mL). Dicyclohexyl carbodiimide (DCC; 0.21 g, 1.0 mmol) and *N* hydroxysuccinimide (NHS; 0.12 g, 1.0 mmol) were added. The solution was stirred under N₂ in the dark at 25 °C for 12 h see Fig. 2. All data was in agreement with previously reported literature [25].

**Fig. 3:**
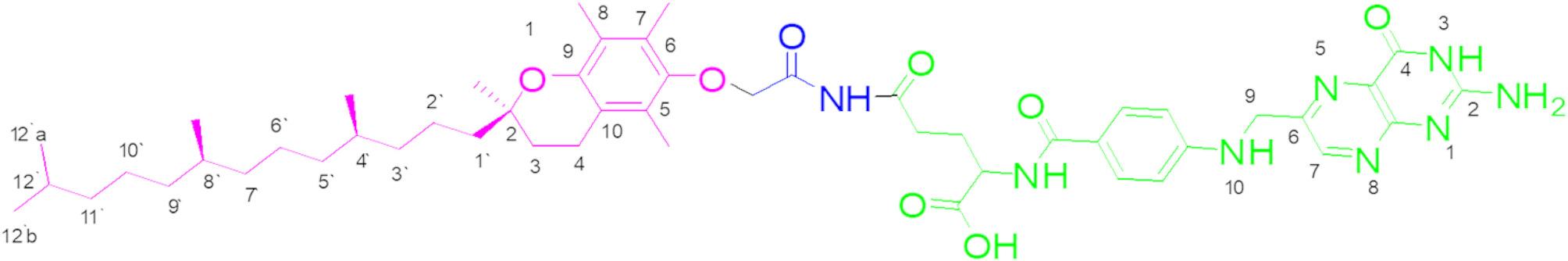
Shows the conjugation of α-Tocopherol with folic acid through a peptide bond

Conjugation: The amino-derivatized vitamin E (dissolved in 10 mL CHCl₃, diluted with 5 mL DMSO) was added dropwise to the activated folic acid mixture. The reaction was stirred under N₂ in the dark at 25 °C for 24 h. Purification of Conjugate: Chloroform was removed by rotary evaporation at 35 °C. The residue was treated with diethyl ether (3 × 20 mL) to extract the conjugate from DMSO. The combined ether extracts were concentrated *in vacuo.* The crude product was recrystallized from DMSO/H₂O (1:5 v/v) to afford the conjugate as a crystalline solid. The final product as seen in Fig. 3 has a melting point of more than 300 °C, physical state is a pale Brown crystalline solid, and a yield of 0.55 g (60%) as distinguishing features. The effective synthesis of the α-Tocopherol-folic acid conjugation is confirmed by characterization methods including spectroscopic data (¹H NMR, FT-IR), which offer comprehensive structural details about the conjugated product. With the help of this technique, folic acid and α-Tocopherol can be effectively conjugated, which may increase their bioavailability and therapeutic efficiency.

#### Purification clarifications

Ether Extraction: Diethyl ether selectively partitioned the conjugate away from DMSO-soluble impurities. Recrystallization: DMSO/H₂O solvent pair removed residual coupling reagents (DCC, NHS) and byproducts. Drying: The ether-extracted conjugate was dried under high vacuum (0.1 mmHg) prior to recrystallization. This protocol ensures high-purity conjugate synthesis through optimized phase separation and crystallization, critical for biological applications.

The pale Brown crystals; yield 0.55 g (60%); mp > 300 °C. IR (KBr) νmax 3318, 3126 (NH_2_), 1697.9 (CO- group) cm^–1^. MS m/z (%): 911.1396 [M^+^ + 1, 10). Anal. Calcd. for C_50_H_70_N_8_O_8_: C, 65.91; H, 7.74; N, 12.30. Found C, 65.81; H, 7.63; N, 12.00. The ^1^ H-NMR, ^13^C-NMR data (See Table 1).

**Table 1:** Shows the analysis data from 1H NMR, and 13 C NMR spectroscopy, confirming the successful formation of a peptide bond and the presence of functional groups associated with the α-tocopherol-folate (VAF) conjugate

1H-NMR (DMSO-d6, 400 MHz):	13C-NMR (DMSO-d6, 100 MHz):
δ = 12.50 (1 H, br., COOH); 11.55, 10.50 (2 H, s, NH-benzamide, NH-peptide bond); 8.65 (1 H, s, 7-pteridine group); 7.60–8.10 (3 H, m, Ar-ring); 6.95 (3 H, s, 2-NH2, 3-NH-pteridine moiety, 5.57 (1 H, s, NH -CH-COOH), 4.50, 4.70 (6 H, m, CH2); 4.50 (2 H, s, -OCH2), 2.18 (3 H, s, CH3, 2CH3), 2.89 (3 H, s, CH3, 5-CH3), 1.72 (3 H, m, 3CH, 4ʹ-H, 8ʹ-H, 12ʹ-H), 1.62 (6 H, m, 2CH3, 7-CH3,8-CH3), 1.52 (24 H, m, 11CH2, 3-H, 4-H, H(1ʹ,2ʹ,3ʹ,5ʹ,6ʹ,7ʹ,9ʹ,10ʹ,11ʹ), 1.02 (12 H, m,4CH3,4ʹ-CH3, 8ʹ-CH3, 12aʹ-CH3, 12bʹ-CH3)	δ = 25.0 (CH3, C2), 25.5 (CH3,C7), 25.7 (CH3, C8), 26.0 (C, C12ʹb), 26.5 (C, C12ʹa), 26.7, 28.5 (C, C,3,4), 28.7 (CH2, C10’), 29.6 (CH3, C5), 29.8, 30.4, 30.7 (C, C4ʹ,8ʹ,11ʹ), 32.0, 32.9 (C, C12ʹa), 33.7, 31.2 (C, C8), 33.5 (C, C12ʹ), 35.7, 36.3, 36.4, 37.4, 38.4, 39.3, 40.0 (C, C1ʹ,2ʹ,3ʹ,5ʹ,6ʹ,9ʹ), 47.4, 52.6, 70.0 (CH2,OCH2), 74.8 (C, C2), 112.6, 117.6 (C, C9), 122.6, 124.0 (C, C7’), 126.6 (C, C4’), 128.4, 129.1,129.3 (C, C7,8), 130.6, 157.1, 154.9, 152.7, 150.0, 147.5, 147.8 (C, C6), 148.2 (C, C10), 166.6, 167.5, 170.1, 173.2, 174.4 (C, CO).

### Biology

#### Cell culture

Normal human Lung cells (Wi-38) and human Lung adenocarcinoma cells (A549) were grown in RPMI-1640 medium supplemented with 10% fetal bovine serum (FBS; Gibco), 2 mM l-glutamine, 100 IU/mL penicillin, and 100 µg/mL streptomycin (Lonza, Belgium), which were procured from VACSERA (Egyptian Organization for Biological Products and Vaccines). The cells were maintained in a humidified atmosphere with 5% CO_2_ at 37 °C.

#### Measuring the IC_50_ of the novel VAF conjugate using WST-1 reagent assay

The novel α-tocopherol-folate conjugate (VAF) Stock solution was prepared in concentrations ranging from 2.5 to 60 µM. Cell viability and proliferation were assessed using the cell proliferation reagent WST. Using the procedure outlined by Ishiyama, the cell proliferation reagent WST-1 (Clontech Laboratories, Takara Co., Japan) was employed for this test [26]. This is a colorimetric assay based on the cleavage of the tetrazolium salt WST-1 to formazan by mitochondrial dehydrogenases in metabolically active cells. This assay provides a sensitive, non-radioactive, and quantitative measure of viable cells with the advantage of being performed directly in the culture plate without additional solubilization steps. Briefly, Wi-38 and A549 cells were seeded in 96-well plates at a density of 1 × 10^4^ cells per well and allowed to adhere overnight under standard culture conditions (37 °C, 5% CO₂). Cells were then treated with increasing concentrations of the novel VAF conjugate (2.5 to 60 µM) for 24 h. Following treatment, 10 µL of WST-1 reagent was added to each well, and plates were incubated for 2–4 hours at 37 °C. The formation of the soluble formazan dye was quantified by measuring absorbance at 440 nm with a reference wavelength above 600 nm using a microplate reader. Cell viability was calculated as a percentage relative to untreated control wells [27].

#### Evaluations of cell viability assay

To compare and assess the impact of varying dosages of folic acid, α-tocopherol, and α-Tocopherol- Folate conjugate, the mitochondrial activity of Wi3-38 and A549 cells was measured using WST-1 assay. A 96-well plate was usually used for cell subculturing. The number of cells in each well is 1 × 10^4^, and three distinct concentrations of cells are administered. Every experiment was run three times. The cells were subsequently incubated for an extra night before adding the WST-1 kit reagent and another 24 h were spent incubating the cells.

#### Confocal laser scanning microscopy (CLSM)

With the assistance of Zen 2009 software and a CO_2_ incubator, confocal laser scanning microscope (CLSM, LSM 710 Carl Zeiss, Germany) images of Wi-38 and A549 cells were taken both before and after the treatment experiment. With Zen 2012 (blue edition), processing was carried out. To get pictures, a 40× oil immersion objective was employed. Additionally, the emission profile of α-Tocopherol coupled with Folate was measured using a lambda scan. A coverslip was coated with 20.0 µL of folic acid, α-tocopherol, and folate conjugated to α-tocopherol. Laser lines 405.0, 458.0, 488.0, 514.0, 543.0, and 633.0 nm were used to conduct a lambda scan from 432.0 to 712.0 nm.

### Analytical statistics

The data on cell survival was expressed as the mean (±) standard error (SD), and the significance of the differences from the control group was ascertained using Origin Lab 9. The influence of both test material concentrations was investigated using the one-way ANOVA test to establish statistical significance (*P* < 0.05).

## Results and discussion

### Characterization of the conjugates (VAF)

**Fig. 4:**
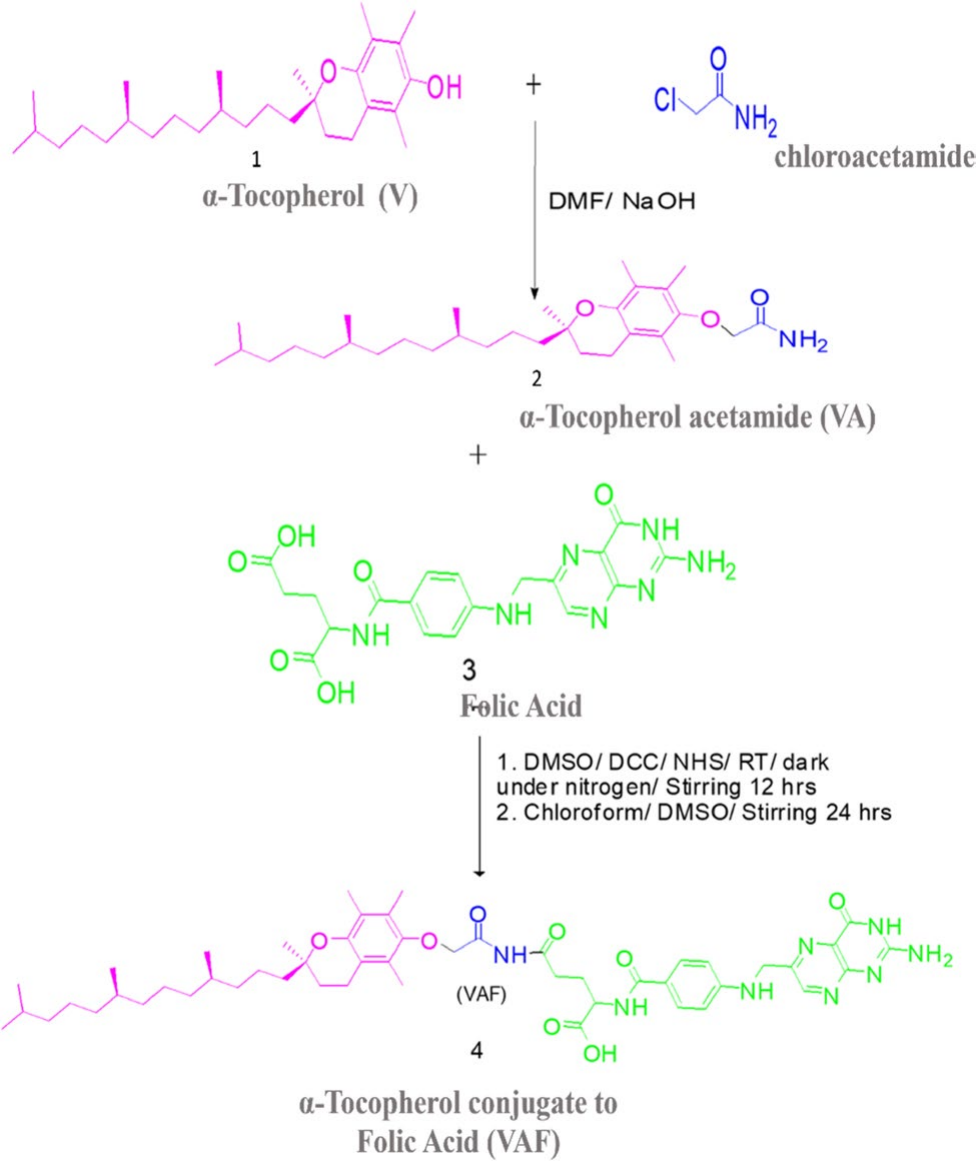
Schematic representation of α-Tocopherol followed by reaction with folic Acid

The combination process mechanism of α-Tocopherol and Folic acid (Fig. 4) can be started with the synthesis of α-Tocopherol acetamide derivative 2 by reacting α-Tocopherol 1 with chloroacetamide in a procedure that was similar but slightly modified, and this was used as a precursor material to synthesize the final target product α-Tocopherol-Folic acid (VAF) conjugate by the coupling reaction of derivative 2 (α-Tocopherol acetamide (VA)) with folic acid (3). The conjugation reaction occurred selectively, one of the carboxyl groups in Folic acid and the amino group of another compound 2 (VA) where water was released to form a targeting compound via peptide bond.

This study investigated the structural characterization of the synthesized folate-α-tocopherol conjugate was comprehensively supported by FTIR and NMR spectroscopy, providing strong evidence for the successful formation of the desired peptide (amide) linkage and the integrity of the molecular framework.

**Fig. 5:**
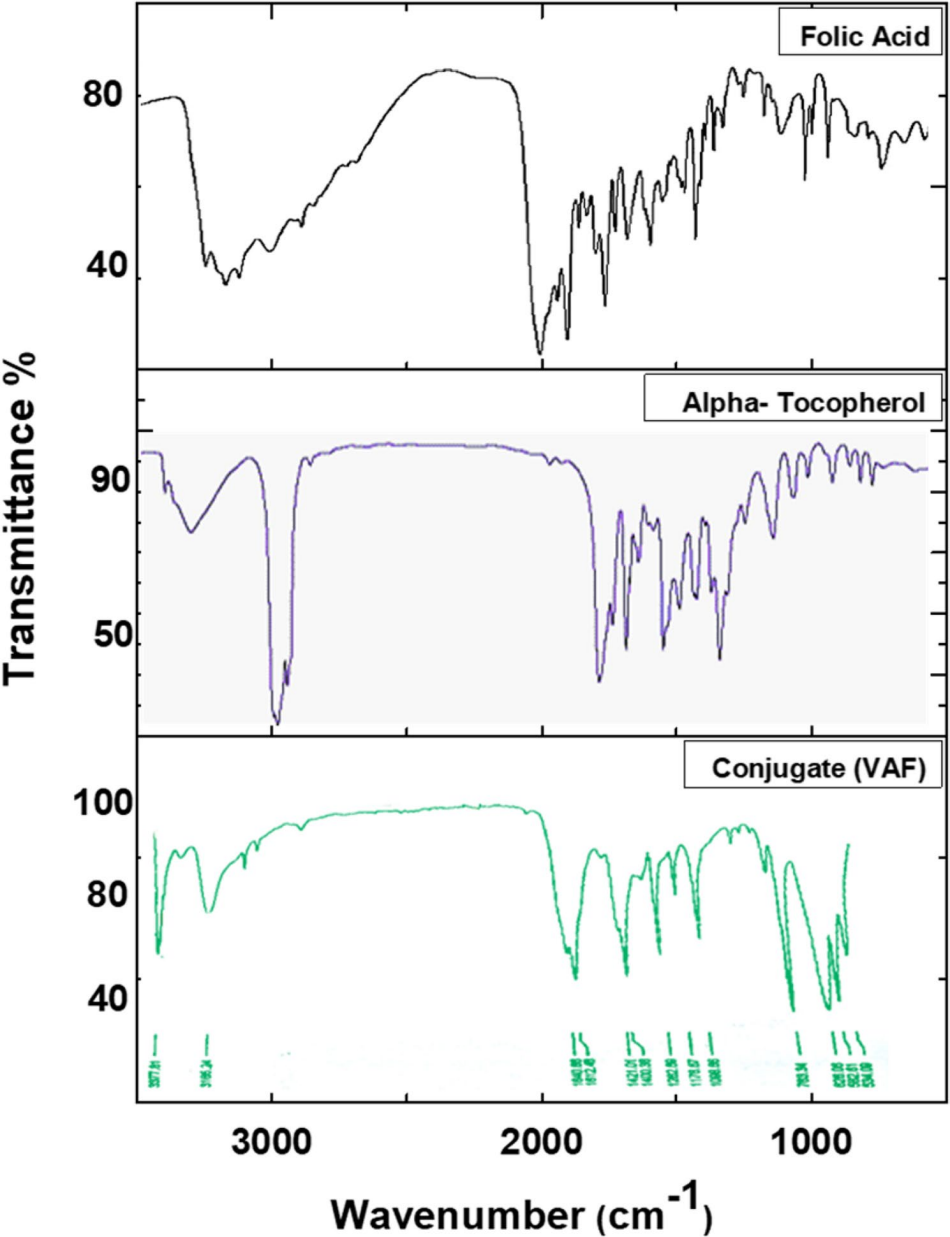
Shows the FTIR spectra of folic acid, α-tocopherol, and α-tocopherol-folate (VAF) conjugate

The FTIR spectrum revealed characteristic absorption bands indicative of peptide bond formation (Fig. 5). The appearance of new absorption bands is indicated in the presence of bands at 3318, 3126, 1640, 1612, 1262, and 763 cm⁻¹. The broad absorption at 3318 cm⁻¹ and the band at 3126 cm⁻¹ are attributed to N–H stretching vibrations, which are typical of amide and amine groups and often signify the presence of peptide bonds in organic molecules. The bands at 1640 and 1612 cm⁻¹ correspond to the amide I region, primarily associated with C = O stretching of the peptide bond, and is a definitive marker for amide formation and protein secondary structure. Additionally, 1262 cm⁻¹; This band is within the amide III region (1250–1350 cm⁻¹), which involves C–N stretching and N–H bending vibrations of the peptide bond. The amide III band is another hallmark of peptide bond presence. However, the 763 cm⁻¹ band is in the fingerprint region and often associated with aromatic C–H out-of-plane bending, it can also be present in compounds containing aromatic rings or substituted amides. So, the presence of these bands, especially those in the N–H stretching (3318, 3126 cm⁻¹), amide I (1612 cm⁻¹), and amide III (1262 cm⁻¹) regions strongly suggests that a peptide (amide) bond has formed in our product. This aligns with the typical FTIR fingerprints of proteins and peptides. These findings are consistent with previous literature, where similar FTIR band assignments have been used to confirm peptide bond formation in synthetic and natural compounds. The observed spectral features not only confirm the successful synthesis of the conjugate but also demonstrate the effectiveness of the applied synthetic strategy [28].

^1^H NMR and ^13^C NMR spectra supported our targeted conjugate (VAF) (Table 1). The ¹H-NMR spectrum (DMSO-d₆, 400 MHz) provided detailed insights into the proton environment of the conjugate. The broad singlet at δ 12.50 ppm corresponds to the carboxylic acid proton (COOH), confirming the presence of free acid groups. Two singlets at δ 11.55 and 10.50 ppm are assigned to the amide protons of the benzamide and peptide bond NH groups, respectively, further corroborating peptide bond formation. The singlet at δ 8.65 ppm corresponds to the proton of the 7-pteridine group of folic acid, while the multiplet between δ 7.60 and 8.10 ppm represents aromatic protons of the benzene ring, consistent with the folate moiety. Signals at δ 6.95 ppm (3 H, singlet) are attributed to the amino protons (2-NH₂ and 3-NH) of the pteridine moiety. The singlet at δ 5.57 ppm corresponds to the NH proton adjacent to the CH–COOH group, indicative of the peptide linkage. Methylene protons (CH₂) are represented as a number at δ 4.50 and 4.70 ppm (6 H), including the unique singlet at δ 4.50 ppm that is attributed to the -OCH₂- group connecting the conjugate components. The complicated multiplets between δ 1.02 and 1.72 ppm correspond to diverse methylene and methyl groups in the tocopherol side chains and linker regions, but the methyl protons show as singlets at δ 2.18 and 2.89 ppm, referring to methyl groups at different places.

The ¹³C-NMR spectrum (DMSO-d₆, 100 MHz) further substantiated the molecular structure. Signals at δ 166.6, 167.5, 170.1, 173.2, and 174.4 ppm correspond to carbonyl carbons (C = O) of amide and carboxylic acid groups, confirming peptide bond formation and the presence of free acid functionalities. Aromatic carbons of the pteridine and benzene rings resonate between δ 112.6 and 150.0 ppm, consistent with the folate moiety. The methylene carbons linked to oxygen (-OCH₂-) appear at δ 70.0 ppm, while multiple signals between δ 25.0 and 40.0 ppm correspond to methyl and methylene carbons of the tocopherol side chains and linker regions. The presence of these signals confirms the integrity of both folic acid and α-tocopherol components within the conjugate [29].

The combined FTIR and NMR spectroscopic data unequivocally confirm the successful synthesis of the folate-α-tocopherol conjugate via peptide bond formation. The FTIR spectra demonstrate characteristic amide bond vibrations, while the detailed ¹H- and ¹³C-NMR spectra provide comprehensive evidence of the conjugate’s chemical structure, including the folate and tocopherol moieties and the peptide linkage connecting them.

### In vitro cytotoxicity

#### IC_50_ of the VAF conjugate

**Fig. 6:**
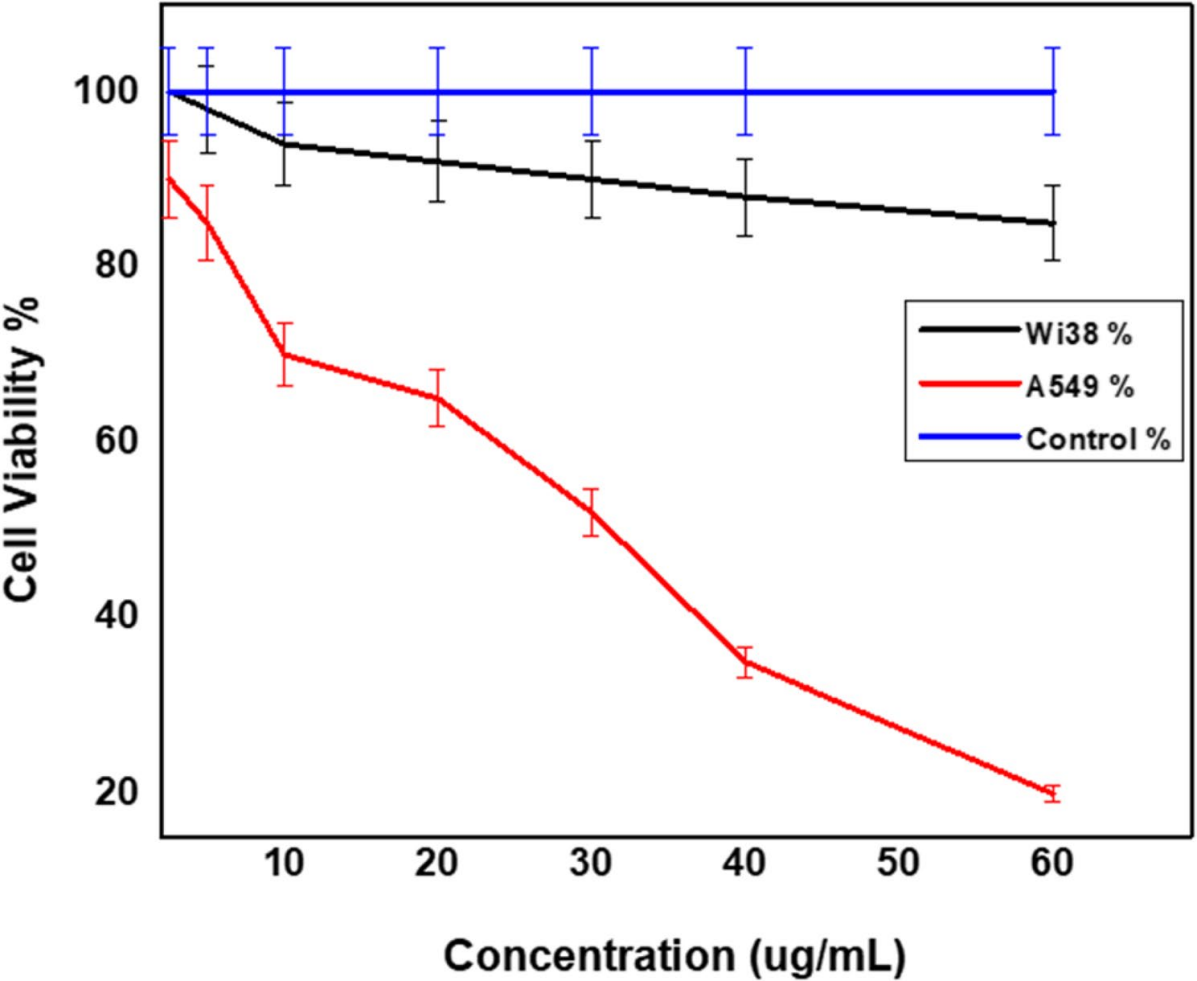
Indicates the effect of the novel (VAF) conjugate on both Wi38 and A549 at concentrations ranges from 2.5 to 60 µg/mL relative to the negative control

The cytotoxic effects of the novel conjugate were evaluated on both normal lung fibroblast cells (Wi-38) and human lung adenocarcinoma cells (A549) across a range of concentrations (2.5 to 60 µM) (Fig. 6). The viability of Wi-38 cells remained relatively high throughout the treatment, with only a modest decrease from 98% at 5 µg/mL to 85% at 60 µg/mL, indicating limited cytotoxicity towards normal cells. In contrast, A549 cancer cells exhibited a pronounced dose-dependent reduction in viability, decreasing significantly from 90% at 2.5 µg/mL to 20% at 60 µg/mL (*p* < 0.01 at concentrations ≥ 20 µg/mL). This differential sensitivity underscores the selective anticancer potential of the conjugate. The calculated IC_50_ value for A549 cells, the concentration at which 50% of cell viability is inhibited, was approximately 30 µg/mL, reflecting potent cytotoxic activity against the cancer cell line. Conversely, Wi-38 cells did not reach an IC_50_ within the tested concentration range, further supporting the conjugate’s preferential toxicity towards malignant cells. Statistical analysis using ANOVA confirmed that the reduction in A549 viability was statistically significant compared to Wi-38 cells at corresponding concentrations (*p* < 0.05). These results demonstrate that the conjugate effectively targets lung cancer cells while sparing normal fibroblasts, highlighting its promise as a selective therapeutic agent [30].

#### Evaluation of cell viability

**Fig. 7:**
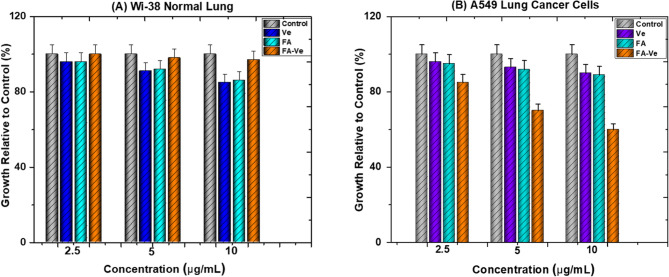
Shows the cytotoxic effect of Folic acid (F), α-Tocopherol (V), and conjugation of Folic acid with α-Tocopherol (VAF) on the (**A**) Wi-38 normal Lung cells as well as (**B**) A549 Lung cancer cells

The conjugate’s structural confirmations justify the synthetic approach and bolster the novel conjugate’s biological evaluation as a targeted therapeutic agent. Figure 7. Shows four different groups; (1) The control group, (2) The α-Tocopherol (V) group, (3) The Folic Acid group (F) group, and (4) The conjugation of α-Tocopherol with Folic acid (VAF) group. The experiment was applied to two types of cells; Normal Lung Cells (Wi-38), and Lung Cancer Cells (A549), separately. Moreover, each compound is studied in three different concentrations (2.5, 5, and 10 µg/mL) for each cell type compared with the control group [31, 32]. This experiment result indicated approximately no effect of the conjugate on the normal lung cells (Wi-38). However, folate and α-Tocopherol have a significant effect on the Wi-38 cells when used individually **(**Fig. 7A**)**. Interestingly, the results of the cytotoxic effect of the conjugates on the A549 cells revealed a significant inhibitory effect with moderate and high concentrations. However, using Folic acid or α-Tocopherol has minor effects on A549 cells under the same conditions (Fig. 7B). This relates to the possibility that conjugating folic acid with α-tocopherol will target lung cancer cells more effectively because these cells have folate receptors [33]. The overexpression of many receptors in cancer cells relative to healthy cells enables drug delivery to tumor cells. This supports the findings of Weitman et al., 1992, who state that the folate receptor, in particular its isoform alpha (FR-α), is a high-affinity membrane-bound protein that is overexpressed on the surface of many malignancies [34, 35]. These tumors include the brain, breast, lung, colon, and kidney, whereas FR expression is absent or present at low levels in healthy cells [36]. This FR-α regulates the uptake of folic acid inside cells [37].

#### Confocal images of the lung cells

**Fig. 8:**
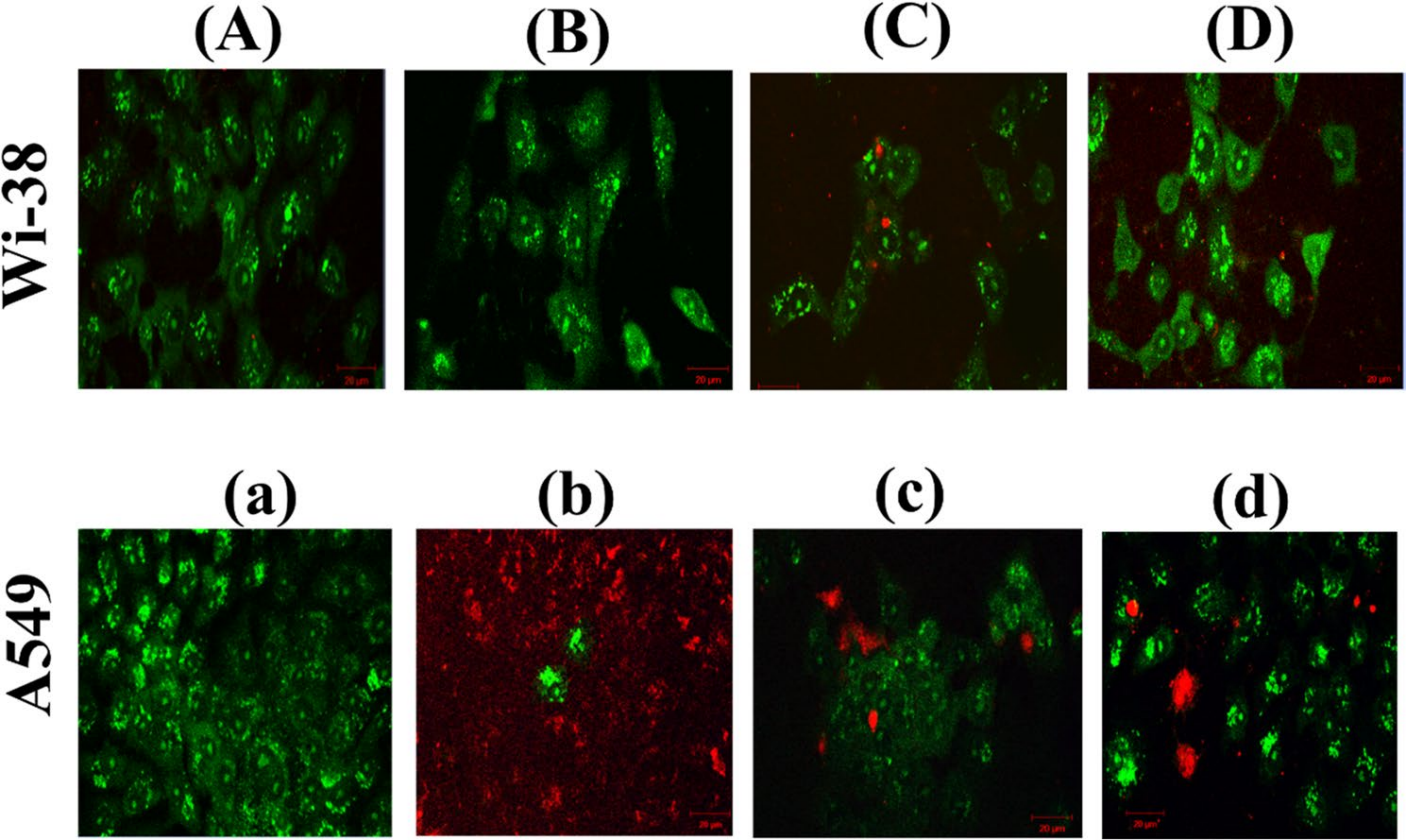
Shows the confocal imaging of live and dead Wi-38 and A549 cells shows the control (**A**, **a**), conjugates (**B**, **b**), Folate (**C**, **c**), and α-Tocopherol (**D**, **d**). Live cells are indicated by green cells stained with acridine orange (AC), while dead cells are indicated by red cells stained with propidium iodide (PI). Scale bars are 20 μm in length

To examine the potential anticancer effects of Folic Acid, α-Tocopherol, and the combination of α-Tocopherol and Folic Acid, CLSM assessed the Wi-38 and A549 cells’ viability. Acridine orange (AO) and propidium iodide (PI), two fluorescent nucleic acid dyes, were utilized to stain the live and dead cells of Wi-38 and A549 cells. Following the use of conjugation (VAF), α-Tocopherol (V), and Folic Acid (F), individually, the stained cells were observed under the CLSM to evaluate the cell viability. Subsequently, k-means clustering and superpixels were proposed as a method to separate and classify the living and dead cells. This technique was developed based on the color properties of the cells as seen in Fig. 8. The observation is that nearly all of the cells in the control group were stained with green, indicating that the cells had the potential to internalize the dye. Owing to the conjugation’s negligible effect on Wi-38, normal lung cells, the conjugate group looks almost exactly like the control group. However, the high dose of F or V had a cytotoxic effect, causing red cells to be visible in Wi-38 cells. On the other hand, the cancer lung cells (A549) treated with the conjugate (VAF) displayed more red cells, up to 66% in comparison to the free folate or Vitamin E treated cells group (85–96%). The appearance of colors red or yellow after PI dye staining is linked to both early and late apoptosis. This indicates many dead cells with broken membranes, which allowed the PI stain to enter the cells [38]. Cellular reactions to V and F at the subcellular level, such as mitochondrial accumulation or modifications in membrane integrity, can be seen using confocal imaging. These findings may shed light on the processes by which these substances cause cytotoxicity. To determine if V and F specifically target cancer cells while preserving healthy tissues crucial component of creating cancer treatments with few adverse effects, we can compare their effects on diseased and normal lung cells.

### Mechanism of action

FRα is a folate receptor isoform. Only cells essential for folate reabsorption or embryonic development express the low expression of folate receptors in normal tissues. However, in low folate levels, cells overexpress folate receptors (FRs) in some pathological diseases, including cancer. This is done to facilitate the cellular uptake of folate [39, 40]. This overexpression renders them potentially therapeutic targets in the management of cancer disorders to obtain a targeted distribution of medications at altered cell levels. Moreover, enhances the effectiveness of the pharmaceutical treatments and lessens their systemic toxicity. FRα, the folate receptor, is the target of this possible anticancer drug. The increased binding affinity of FRα for folate may enhance the folate uptake by cancer cells. The method employed to accomplish this folate receptor targeting is the application of ligands related to the therapeutic drugs (α-Tocopherol) that have a high affinity for FRs (Folic Acid). Interestingly, this conjugate represents a promising anticancer drug delivery.

## Conclusion

This investigation presents a novel; natural conjugate composed of folate and α-tocopherol (VAF). The goal of this combination is to minimize off-target toxicity while selectively targeting cancer cells. The conjugation was successfully confirmed by the structural and physicochemical study, which made it appropriate for use in biological applications. According to cytotoxicity assays, VAF revealed that significantly reduced the viability of cancer cells in a dose-dependent manner while preserving a high level of compatibility with healthy, normal cells. This selective toxicity is explained by the enhanced uptake of the folate-conjugated complex by cancer cells, which overexpress the folate receptor. Over and above that, the antioxidant and pro-apoptotic effects of α-tocopherol. The dual functionality of VAF as both a targeting and therapeutic agent provides a strong base for its advancement into a harmless, more efficient delivery for targeted cancer therapy. Moreover, the integration of natural compounds in the structure responds to the growing demand for environmentally friendly, non-toxic, and economical alternatives to conventional chemotherapeutic agents. The results not only validate the therapeutic potential of VAF but also open up possibilities for incorporating similar natural conjugates into medicine. Future research should concentrate on pharmacokinetics, mechanistic investigation of cell death pathways, and in vivo evaluation to further validate its safety and effectiveness for clinical use.

## Supplementary Information

Supplementary Material 1

Supplementary Material 2

## Data Availability

The data produced and analyzed during the current study are included within the article and its additional files.

## Abbreviations

VA: α-Tocopherol Acetamide
VAF: Conjugation of α-Tocopherol with Folic Acid
FA: Folic Acid
NO_2_: Nitrogen Dioxide
A549: Cancer Lung cells
Wi-38: Healthy Lung cells
DCC: Dicyclohexyl Carbodiimide
NHS: n-Hydroxy Succinimide
FT-IR: Fourier Transform Infrared
NTNU: Norwegian University of Science and Technology
NaOH: Sodium Hydroxide
CHCl_3_: Chloroform
VACSERA: Egyptian Organization for Biological Products and Vaccines
CO_2_: Carbon Dioxide
CLSM: Confocal Laser Scanning Microscopy
AO: Acrylidine Orange
PI: Propidium Iodide
FRs: Folate Receptors

## References

[1] Brown JS, Amend SR, Austin RH, Gatenby RA, Hammarlund EU, Pienta KJ. Updating the definition of cancer. Mol Cancer Res. 2023;21(11):1142–7. 37409952 10.1158/1541-7786.MCR-23-0411 PMC10618731

[2] Thandra KC, Barsouk A, Saginala K, Aluru JS, Barsouk A. Epidemiology of lung cancer. Contemp Oncol (Pozn). 2021;25(1):45–52. 33911981 10.5114/wo.2021.103829 PMC8063897

[3] Bowman B, Russell R, ILSI Press’ International Life Sciences Institute. Present Knowledge in Nutrition, 9th ed. Washington, DC. ISBN 1 57 881 199 1 98. 2006(3): 187.

[4] Thabet RH, Alessa REM, Al-Smadi ZKK et al. (2024) ‘Folic acid: friend or foe in cancer therapy’. Journal of International Medical Research. 2024;52(1). 10.1177/03000605231223064 PMC10935767 38229460

[5] Cheung A, Bax HJ, Josephs DH, Ilieva KM, Pellizzari G, Opzoomer J, et al. Targeting folate receptor alpha for cancer treatment. Oncotarget. 2016;7(32):52553–74. 27248175 10.18632/oncotarget.9651 PMC5239573

[6] Lohade AA, Jain RR, Iyer K, Roy SK, Shimpi HH, Pawar Y, et al. A novel folate-targeted nanoliposomal system of doxorubicin for cancer targeting. AAPS PharmSciTech. 2016;17:1298–311. 26689406 10.1208/s12249-015-0462-2

[7] Gao W, Xiang B, Meng TT, Liu F, Qi XR. Chemotherapeutic drug delivery to cancer cells using a combination of folate targeting and tumor microenvironment-sensitive polypeptides. Biomaterials. 2013;34(16):4137–49. 23453200 10.1016/j.biomaterials.2013.02.014

[8] Xiang G, Wu J, Lu Y, Liu Z, Lee RJ. Synthesis and evaluation of a novel ligand for folate-mediated targeting liposomes. J Pharm. 2008;356(1):29–36. 10.1016/j.ijpharm.2007.12.030 PMC2424191 18258394

[9] Yang C, Chen H, Zhao J, Pang X, Xi Y, Zhai G. Development of a folate-modified Curcumin loaded micelle delivery system for cancer targeting. Colloids Surf B Biointerfaces. 2014;121:206–13. 24984268 10.1016/j.colsurfb.2014.05.005

[10] Wang X, Quinn PJ. Vitamin E and its function in membranes. Prog Lipid Res. 1999;38:309–36. 10793887 10.1016/s0163-7827(99)00008-9

[11] Cho E, et al. Intakes of vitamins A, C, and E and folate and multivitamins and lung cancer: a pooled analysis of 8 prospective studies. Int J Cancer. 2006;118:970–8. 16152626 10.1002/ijc.21441

[12] Donnelly J, Appathurai A, Yeoh HL, Driscoll K, Faisal W. ‘Vitamin E in Cancer Treatment: A Review of Clinical Applications in Randomized Control Trials.’ Nutrients. 2022;14(20):4329. 36297013 10.3390/nu14204329 PMC9611110

[13] Cooney RV, Franke AA, Harwood PJ, Hatch-Pigott V, Custer LJ, Mordan LJ. Gamma-tocopherol detoxification of nitrogen dioxide: superiority to alpha-tocopherol. Proc Natl Acad Sci U S A. 1993;90:1771–5. 8446589 10.1073/pnas.90.5.1771 PMC45961

[14] Smolarek AK, Suh N. Chemopreventive activity of vitamin E in breast cancer: a focus on γ- and δ-tocopherol. Nutrients. 2011;3:962–98. 22254089 10.3390/nu3110962 PMC3257724

[15] Kamal-Eldin A, Appelqvist LA. The chemistry and antioxidant properties of tocopherols and tocotrienols. Lipids. 1996;31:671–701. 8827691 10.1007/BF02522884

[16] Cillard J, Cillard P. Prooxidant effect of alpha-tocopherol on essential fatty acids in aqueous media. Ann Nutr Aliment. 1980;34:579–91. 7469263

[17] Lee IM, Cook NR, Gaziano JM, Gordon D, Ridker PM, Manson JE, et al. Vitamin E in the primary prevention of cardiovascular disease and cancer: the women’s health study: a randomized controlled trial. JAMA. 2005;294:56–65. 15998891 10.1001/jama.294.1.56

[18] O’Shannessy DJ, Yu G, Smale R, Fu YS, Singhal S, Thiel RP, et al. Folate receptor alpha expression in lung cancer: diagnostic and prognostic significance. Oncotarget. 2012;3(4):414–25. 22547449 10.18632/oncotarget.519 PMC3380576

[19] Nawaz FZ, Kipreos ET. Emerging roles for folate receptor FOLR1 in signaling and cancer. Trends Endocrinol Metabolism. 2022;33(3):159–74. 10.1016/j.tem.2021.12.003 PMC8923831 35094917

[20] Gonzalez T, Muminovic M, Nano O, Vulfovich M. Folate receptor alpha—a novel approach to cancer therapy. Int J Mol Sci. 2024;25(2):1046. 38256120 10.3390/ijms25021046 PMC11154542

[21] Peer D, Karp JM, Hong S, Farokhzad OC, Margalit R, Langer R. Nanocarriers as an emerging platform for cancer therapy. Nat Nanotechnol. 2007;2(12):751–60. 18654426 10.1038/nnano.2007.387

[22] Alexeree SMI. (2025). Photodynamic therapy based on curcumin-mediated nanomedicines for wound healing. *Recent Advances in Nanomedicines Mediated Wound Healing*. Elsevier; 459–470.

[23] Low PS, Kularatne SA. Folate-targeted therapeutic and imaging agents for cancer. Curr Opin Chem Biol. 2009;13(3):256–62. 19419901 10.1016/j.cbpa.2009.03.022

[24] Koo H, Huh MS, Sun IC, Yuk SH, Choi K, Kim K, Kwon IC. Nanotechnology for targeted cancer therapy and imaging. Adv Drug Deliv Rev. 2022;64(2):114–25.

[25] Abdel-Hafez SHH, Gobouri AA, ALShanbari NA, Gad Elrab SMF. Synthesis of novel vitamin E containing Sulfa drug derivatives and study their antibacterial activity. Med Chem Res. 2018;27:2341.

[26] Ishiyama M, Miyazono Y, Sasamoto K, Ohkura Y, Ueno K. A highly water-soluble disulfonated tetrazolium salt as a chromogenic indicator for NADH as well as cell viability. Talanta. 1997;44:1299–305. 18966866 10.1016/s0039-9140(97)00017-9

[27] Alexeree SMI, ElZorkany H, Abdel-Salam Z, Harith MA. A novel synthesis of a chlorophyll b-gold nanoconjugate used for enhancing photodynamic therapy: in vitro study. Photodiagn Photodyn Ther. 2021;35:102444. 10.1016/j.pdpdt.2021.102444 34284147

[28] Rajeshkumar RR, Pavadai P, Panneerselvam T, et al. Enhanced delivery of retinoic acid to breast cancer cells by folate receptor-targeted folic acid-conjugated glutenin nanoparticles for promising treatment of breast cancer. J Polym Environ. 2024;32:2120–39.

[29] Barar J, Kafil V, Majd MH, et al. Multifunctional mitoxantrone-conjugated magnetic nanosystem for targeted therapy of folate receptor-overexpressing malignant cells. J Nanobiotechnol. 2015;13:26. 10.1186/s12951-015-0083-7 PMC4387580 25880772

[30] Yoshino H, Tadano K, Omiya C, et al. Involvement of cellular senescence in the effect of X-irradiated human lung fibroblast WI-38 cells on human lung cancer A549 cell clonogenic potential. Radiat Prot Dosimetry. 2024;200:1608–14. 39540500 10.1093/rpd/ncae089

[31] O’Shannessy DJ, Somers EB, Albone E, Cheng X, Park YC, Tomkowicz BE, Hamuro Y, Kohl TO, Forsyth TM, Smale R, Fu YS, Nicolaides NC. Characterization of the human folate receptor alpha via novel antibody-based probes. Oncotarget. 2011;2:1227–43. 22204844 10.18632/oncotarget.412 PMC3282080

[32] Hassan M, Khamis G, Zorkany H, Alexeree SMI. Innovative approaches to enhancing tamarind seed germination and phytochemical production through laser irradiation: implications for photodynamic therapy. Lasers Med Sci. 2025;40:290. 40537523 10.1007/s10103-025-04527-3 PMC12178999

[33] Abd Elhameed HAH, Attia SM, Mohamed AA, Alexeree SMI, Behery E, Alagawany EI, Cerbo FMR, Azzam A, Mawed MM S. A. The role of Phthalocyanine-Gold nanoconjugates (Pc-Au NCs) in ameliorating the hepatic and renal Toxicity-Induced by silver nanoparticles (Ag NPs) in male rats. Biol Trace Elem Res. 2024;202:5637–52. 38739260 10.1007/s12011-024-04209-1

[34] Weitman SD, Lark RH, Coney LR, Fort DW, Frasca V, Zurawski VR, Kamen BA. Distribution of the folate receptor GP38 in normal and malignant cell lines and tissues. Cancer Res. 1992;52:3396–401. 1596899

[35] Alexeree SMI, Harith MA. (2023) Monitoring the cytotoxic effect of novel nanoconjugates during and after in-vivo photodynamic therapy, AIP Conference Proceedings 2620, 060001.

[36] Shen J, Hu Y, Putt KS, Singhal S, Han H, Visscher DW, Murphy LM, Low PS. (2017). Assessment of folate receptor alpha and beta expression in selection of lung and pancreatic cancer patients for receptor targeted therapies. Oncotarget. 15;9(4):4485–4495. 10.18632/oncotarget.23321 PMC5796989 29435118

[37] Weitman SD, Weinberg AG, Coney LR, Jennings ZVR, Kamen DS B. A. Cellular localization of the folate receptor: potential role in drug toxicity and folate homeostasis. Cancer Res. 1992;52:6708–11. 1330299

[38] Alexeree SMI, Abou-Seri HM, El-Din HES, Youssef D, Ramadan MA. Green synthesis of silver and iron oxide nanoparticles mediated photothermal effects on *Blastocystis hominis*. Lasers Med Sci. 2024;39(1):43–55. 38246979 10.1007/s10103-024-03984-6 PMC10800310

[39] Chen C, et al. Structural basis for molecular recognition of folic acid by folate receptors. Nature. 2013;500:486–9. 23851396 10.1038/nature12327 PMC5797940

[40] Kelemen LE. The role of folate receptor α in cancer development, progression and treatment: cause, consequence or innocent bystander? Int J Cancer. 2006;119(2):243–50. 16453285 10.1002/ijc.21712

