# Supplementary material for: Evaluating the anticancer properties of VAF: a novel folate-α-tocopherol conjugate against lung cancer cells
Source: BMC Cancer. 2025 Sep 16;25:1423. doi: 10.1186/s12885-025-14954-8 (PMC12442261; doi:10.1186/s12885-025-14954-8)
Supplement: Supplementary file 1 — Supplementary Material 1 [file 12885_2025_14954_MOESM1_ESM.pdf]

Dr. Shams
Sample SH-V/Folic DMSO

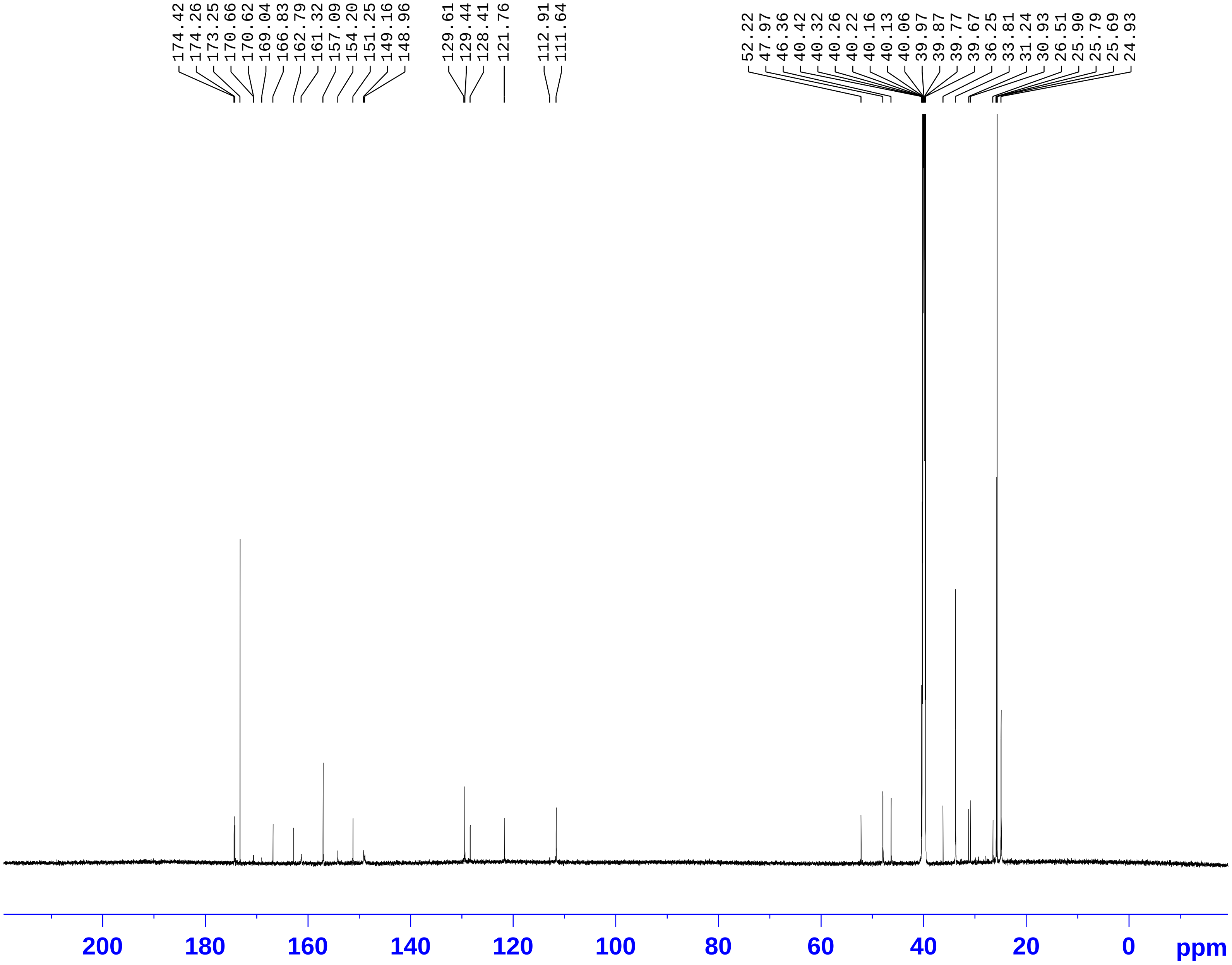

Current Data Parameters
NAME SHAMS SH-V 17-01-2021
EXPNO 11
PROCNO 1

F2 - Acquisition Parameters
Date_ 20210117
Time 19.27
INSTRUM spect
PROBHD 5 mm CPQCI 1H-
PULPROG zgpg30
TD 65536
SOLVENT DMSO
NS 4096
DS 4
SWH 51020.406 Hz
FIDRES 0.778510 Hz
AQ 0.6422528 sec
RG 186.93
DW 9.800 usec
DE 18.00 usec
TE 298.0 K
D1 2.00000000 sec
D11 0.03000000 sec
TD0 1

======== CHANNEL f1 ========
SFO1 213.7917636 MHz
NUC1 13C
P1 45.00 usec
PLW1 12.00000000 W

======== CHANNEL f2 ========
SFO2 850.1534006 MHz
NUC2 1H
CPDPRG[2 waltz16
PCPD2 80.00 usec
PLW2 16.70000076 W
PLW12 0.16700000 W
PLW13 0.10688000 W

F2 - Processing parameters
SI 32768
SF 213.7703875 MHz
WDW EM
SSB 0
LB 1.00 Hz
GB 0
PC 1.40
