# Supplementary material for: Evaluating the anticancer properties of VAF: a novel folate-α-tocopherol conjugate against lung cancer cells
Source: BMC Cancer. 2025 Sep 16;25:1423. doi: 10.1186/s12885-025-14954-8 (PMC12442261; doi:10.1186/s12885-025-14954-8)
Supplement: Supplementary file 2 — Supplementary Material 2 [file 12885_2025_14954_MOESM2_ESM.pdf]

Dr. Shams
Sample SH-V/Folic DMSO

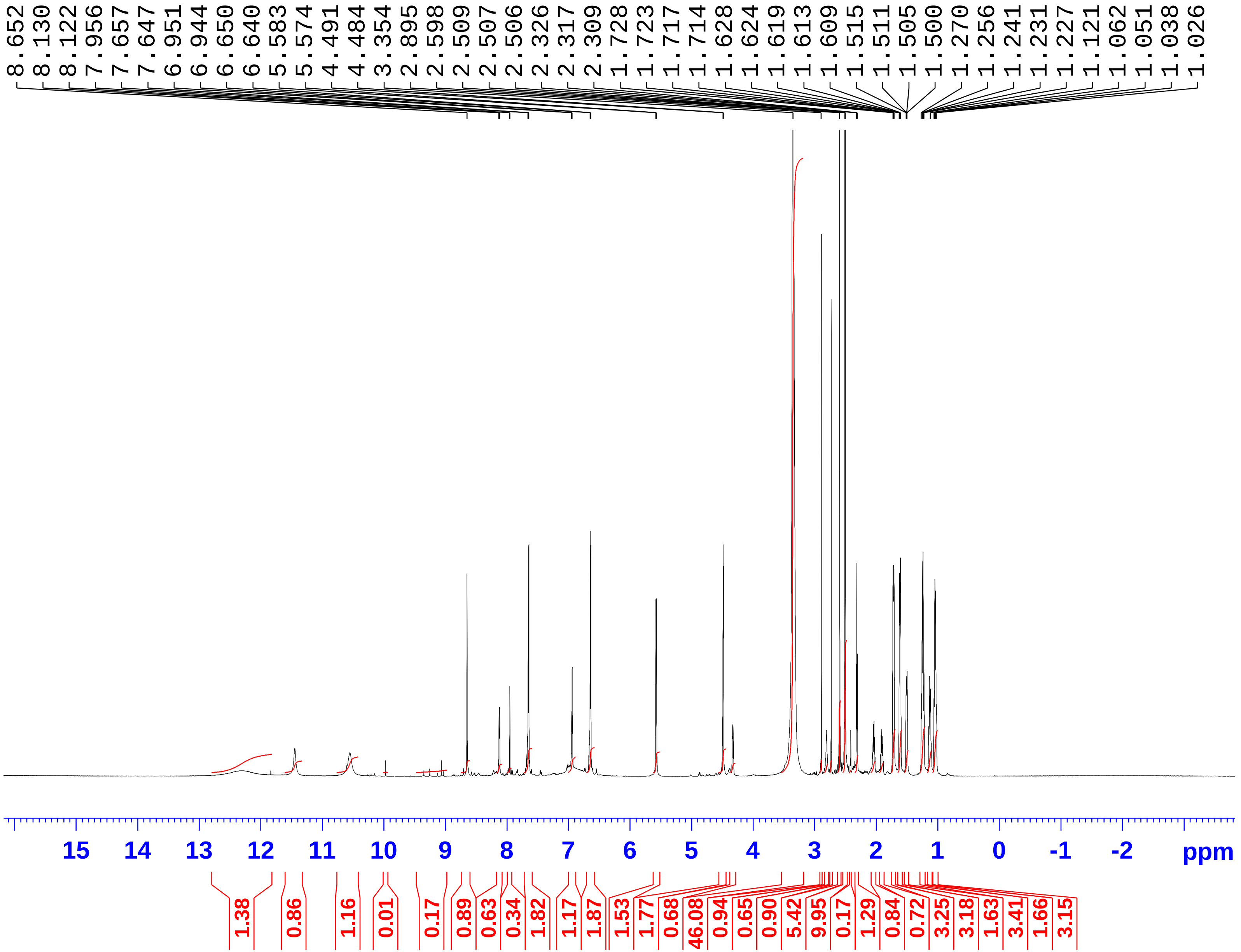

BRUKER

Current Data Parameters
NAME SHAMS SH-V 17-01-2021
EXPNO 10
PROCNO 1

F2 - Acquisition Parameters
Date_ 20210117
Time 16.21
INSTRUM spect
PROBHD 5 mm CPQCI 1H-
PULPROG zg30
TD 65536
SOLVENT DMSO
NS 32
DS 2
SWH 17006.803 Hz
FIDRES 0.259503 Hz
AQ 1.9267584 sec
RG 11.37
DW 29.400 usec
DE 10.00 usec
TE 298.0 K
D1 1.00000000 sec
TD0 1

======== CHANNEL f1 ========
SFO1 850.1552500 MHz
NUC1 1H
P1 8.00 usec
PLW1 16.70000076 W

F2 - Processing parameters
SI 65536
SF 850.1500000 MHz
WDW EM
SSB 0
LB 0.30 Hz
GB 0
PC 2.00
